# Metabolomics-Guided Elucidation of Plant Abiotic Stress Responses in the 4IR Era: An Overview

**DOI:** 10.3390/metabo11070445

**Published:** 2021-07-08

**Authors:** Morena M. Tinte, Kekeletso H. Chele, Justin J. J. van der Hooft, Fidele Tugizimana

**Affiliations:** 1 Department of Biochemistry, University of Johannesburg, Auckland Park, Johannesburg 2006, South Africa; morenatinte@gmail.com (M.M.T.); ckekeletso@gmail.com (K.H.C.); 2 Bioinformatics Group, Wageningen University, 6708 PB Wageningen, The Netherlands; 3 International Research and Development Division, Omnia Group, Ltd., Johannesburg 2021, South Africa

**Keywords:** abiotic stress, metabolomics, 4IR technologies, automation, machine learning

## Abstract

Plants are constantly challenged by changing environmental conditions that include abiotic stresses. These are limiting their development and productivity and are subsequently threatening our food security, especially when considering the pressure of the increasing global population. Thus, there is an urgent need for the next generation of crops with high productivity and resilience to climate change. The dawn of a new era characterized by the emergence of fourth industrial revolution (4IR) technologies has redefined the ideological boundaries of research and applications in plant sciences. Recent technological advances and machine learning (ML)-based computational tools and omics data analysis approaches are allowing scientists to derive comprehensive metabolic descriptions and models for the target plant species under specific conditions. Such accurate metabolic descriptions are imperatively essential for devising a roadmap for the next generation of crops that are resilient to environmental deterioration. By synthesizing the recent literature and collating data on metabolomics studies on plant responses to abiotic stresses, in the context of the 4IR era, we point out the opportunities and challenges offered by omics science, analytical intelligence, computational tools and big data analytics. Specifically, we highlight technological advancements in (plant) metabolomics workflows and the use of machine learning and computational tools to decipher the dynamics in the chemical space that define plant responses to abiotic stress conditions.

## 1. Introduction—A Dawn of a New Era and a Prime to Plant Defenses

### 1.1. The Fourth Industrial Revolution (4IR) Era

**Figure 1:**
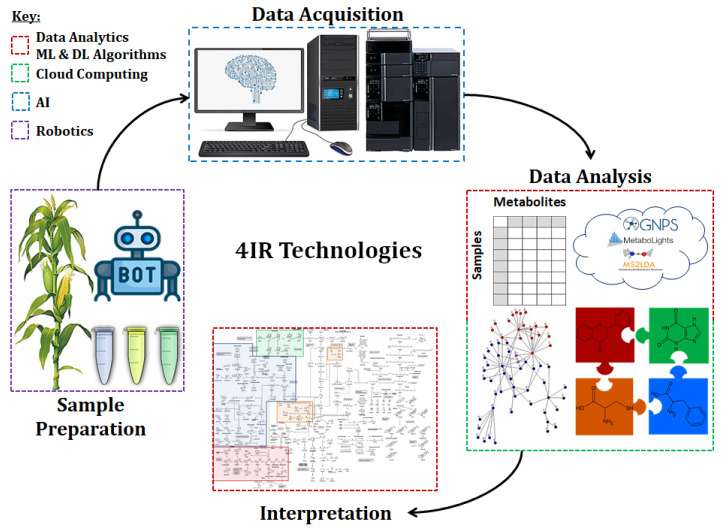
4IR technologies in the plant metabolomics workflow. The 4IR technologies and their implementation within the plant metabolomics workflows are indicated based on colour, as highlighted by the key. An illustration of the preparation of samples with the assistance of robotics, advancements in analytical platforms, equipped with A/AI for sample analysis. The generated (big) data can be uploaded on cloud-based servers, e-infrastructures for data analysis, storage and sharing. Some of these web-based suits include MetaboAnalyst, XCMS Online, MetExplore, PhenoMeNal and GNPS. Computational tools in these *e*-infrastructures involve the use of chemometrics methods, ML and DL algorithms. Metabolic pathway reconstruction and network analysis are often used for biological interpretation of metabolomics data. The IoT is an indispensable component supporting most of these cloud metabolomics frameworks.

The Fourth Industrial Revolution (4IR) era entails the integration of advanced technologies in the physical, digital and biological domains. This includes the confluence and convergence of emerging technologies such as artificial intelligence (AI), the Internet of Things (IoT), big data analytics, cloud computing, robotics and wireless telecommunications [1,2]. These innovative technologies have brought about paradigm shifts and are disruptively boosting many industries globally by encouraging new models that enable the acquisition, sharing, and use of data and resources to produce improved products/services in a faster, cheaper, more effective and sustainable manner [3]. In life sciences, particularly in the field of metabolomics—a multidisciplinary omics science that studies metabolism (Section 2.1)—some of these 4IR technologies have been and are integral components of metabolomics workflows. It suffices to highlight the use of analytical platforms that are equipped with analytical and artificial intelligence (A/AI), generation of big data, application and development of big data analytics involving the use of machine and deep learning (ML and DL) algorithms (Figure 1).

As the field matures, with advancements in technologies, development and applications of state-of-the-art bioinformatics and computational tools, equipped with ML algorithms, are gaining momentum for data mining and interpretation [4,5]. Typical widely adopted examples include the Global Natural Product Social Molecular Networking (GNPS), an ecosystem of tandem mass spectrometry (MS/MS) data storage, analysis and sharing [6], MetaboLights, a cloud computing based repository that enables the sharing and re-use of data and meta-data [7], MS2LDA, a software tool that extracts co-occurring mass fragments and neutral losses from MS/MS spectra using an unsupervised ML algorithm [8], MetaboAnalyst, a web-based service consisting of modules for data pretreatment, mining and pathway analysis, XCMS, a cloud-based data analysis suite for preprocessing untargeted liquid chromatography-mass spectrometry (LC-MS) data, statistical analysis, pathway analysis and multi-omic data integration, MetExplore, an environment for the curation of metabolic networks, and PhenoMeNal, a e-infrastructure with a collection of workflows and tools for metabolomics analysis pipelines [5] (Figure 1). The digitization of mass spectra to aid in biological interpretation of plant metabolomics data is illustrated in a study of more than 70 *Rhamnaceae* plant extracts [9] where the authors illuminate clade-specific chemical signatures annotated through an integrative computational metabolomics workflow.

The increasing momentum and use of 4IR technologies in life sciences, particularly in plant metabolomics, which is the focus of this review, is redefining the ideological boundaries of research and applications in the field. Recent advances in generating comprehensive biological (metabolomics) datasets at high throughput, in combination with enhanced capabilities to mine and interpret these datasets are increasingly allowing scientists to derive a comprehensive understanding for crop plants under consideration [10,11]. Such accurate and predictive models that describe the metabolism of plants under specific conditions would provide novel insights that identify key biological bottlenecks in regard to plant growth and productivity [11,12].

This review focuses on the use of metabolomics in interrogating plant responses to adverse environmental conditions, with a particular attention to the 4IR technologies in this multidisciplinary omics science. Metabolomics is increasingly enabling the decoding of the language of cells at molecular level, advancing the understanding of regulatory network rules and mechanistic events at cellular and chemical space of the plant under consideration. Plants are naturally sessile organisms, and are thus susceptible to changing environmental conditions such as abiotic stress factors that include drought, salinity, extreme high and low temperatures, heavy metals, light and radiation [13,14]. These abiotic stress factors can negatively affect plant growth, development and productivity, and subsequently the agricultural yield. It is, therefore, imperative to comprehensively and predictively understand the plant metabolism under abiotic stresses, as such fundamental and actionable insights (adding to the current knowledgebase, Section 1.2) will contribute to the development of plants with enhanced resilience and productivity, and support strategies that promote plant growth under abiotic stress conditions [15,16]. To logically articulate these aspects, the review is structured to comprise four main components. In Section 1.1. and 1.2, the 4IR era is briefly defined and introduced as well as the current models of plant defense mechanisms. The second main section then elaborates on 4IR technologies in the context of (plant) metabolomics workflows, from sample preparation step to the annotation of metabolites. Automation and technological advancements in analytical techniques, the use of machine learning and computational tools to aid in deciphering the dynamics in the chemical space that define plant defense responses are highlighted. The application of metabolomics to decode plant responses to adverse environmental conditions is increasing, and this is highlighted in the third main section. The review concludes with the fourth main section that contains an outlook on expected developments in the plant metabolomics, driven by advancements of 4IR technologies.

### 1.2. Plant Defense Mechanisms—Current Models

Evolutionally, plants have developed eminently intricate immune systems and defense mechanisms to respond to biotic and abiotic stresses [17]. These responses are stress-dependent, at cellular and molecular levels, but there are also some overlaps in biochemical and physiological events that define plant response to stresses [18,19]. Over the years, various studies have generated a substantial knowledge-base and understanding of plant responses to different adverse environmental stresses, formulating models that explain mechanistic events that govern plant responses [20,21,22]. It suffices here to highlight some of the elucidated molecular events that define plant defense mechanisms, involving the danger perception to activation of downstream molecular and cellular phenomonologies [23]. Perception of abiotic stresses generally results in the generation of reactive oxygen species (ROS) that act as early signaling molecules. Elevated levels of ROS act as a signaling wave that is involved in a defense-related reconfiguration of the hormonal network comprising abscisic acid (ABA), gibberellins (GAs), auxins, jasmonic acid (JA), salicylic acid (SA) ethylene, cytokinins (CKs) and brassinosteroids (BRs). These phytohormones are primary signaling molecules that trigger the expression of stress-related genes and induction of metabolic reprogramming and physiological changes that result in abiotic stress tolerance or resistance [16,21]. The outcome is determined by the directional shift of metabolic reconfigurations and fluxes—either towards an irreversible damage by the stress factor or effective stress resistance and acclimation of the plants. Some of the (general) negative impacts of abiotic stresses on the plant physiology include a reduction in both transduction and photosynthesis rates, a decrease in stomatal conductance and in leaf water content and, subsequently, a reduced growth rate [24,25].

The current understanding of the stress signaling and responses is still the tip of an iceberg. Comprehensive predictive models that describe the activation of different signals, sensing mechanisms, downstream changes in gene expression, metabolism, physiology, growth and development are still lacking. Systems biology approaches, particularly omics sciences (genomics, transcriptomics, proteomics and metabolomics) carried out separately or in integrated manner, hold unique opportunities to generate novel insights describing, comprehensively and predictively the metabolism of plants under specific conditions [10,11,12]. The plant metabolome can echo the effect of environmental stress conditions, and therefore, metabolomics can be applied to provide a snapshot of the plant metabolism at a cellular and molecular level by monitoring the changes in metabolite levels and fluxes, which reflect the biological and physiological processes in response to the stressful conditions [26,27]. Thus, the focal point of this review is the application and potential of metabolomics with 4IR-inspired tools and technologies to aid in elucidating plant defense responses to abiotic stress conditions by illuminating the plant specialized metabolome [28,29,30].

## 2. 4IR Technologies and Plant Metabolomics

Metabolomics is classically defined as an omics science that aims at the analysis of the entire complement of small molecular weight molecules, namely metabolites (≤1500 Da in size), within a biological system under given physiological conditions [29,31]. Metabolomics incorporates the domains of biology, chemistry, chemometric, statistics and computer science. This multidisciplinary scientific field has matured over the last two decades and has gained popularity, particularly in the life sciences, and is becoming indispensable in interrogating cellular biochemistry and elucidating the mechanisms responsible for metabolic changes in response to different physiological conditions [5,32,33,34,35]. In plant sciences, metabolomics has been successfully applied in a broad spectrum of studies including metabolic pathway studies [29], relating genotype and biochemical phenotype [36], silent phenotype mutations [37], plant-environment interactions [27] and plant priming, a phenomenon that pre-conditions plants for enhanced defense against stresses [38]. Metabolomics, particularly for large scale studies, is, however, limited by various bottlenecks; hence, the focus to overcome these challenges has intensified through the development of computational metabolomics tools and improved technologies [9], most of which are 4IR-driven (Figure 1) and are articulated in the following sub-sections on 4IR technologies in metabolomics workflows.

### 2.1. Automation in Sample Preparation

Sample preparation is a key aspect of metabolomic studies, as it is responsible for removing proteins and cellular debris from the metabolites of interest. An ideal sample preparation method should maintain sample integrity, and be simple and robust. Traditional sample preparation steps involve the quenching of the metabolism, cellular lysis with organic solvents or detergents, sonication and centrifugation. The preparation of large numbers of samples, utilizing such sample preparation strategies is, however, time consuming and likely to introduce unwanted variation [39,40]. The demand for automated sample preparation strategies (Figure 1) that will diminish human error, improve reproducibility, decrease extraction time and increase throughput for large-scale metabolomics studies is thus evident [41]. Recently, novel automated sample preparation methods (Table 1) have emerged in the field of metabolomics. These methods are solid-phase extraction (SPE), solid-phase microextraction (SPME), liquid-phase microextraction (LPME) methods (dispersive liquid–liquid microextraction (DLLME), hollow fiber liquid–liquid microextraction (HF-LLME), single drop microextraction (SDME) and the recent electromembrane extraction (EME)), accelerated solvent extraction (ASE), supercritical fluid extraction (SFE), microwave-assisted extraction (MAE) and ultrasound-assisted extraction (UAE) [40,42,43].

The detailed SPME workflow is described in [50]. This method eliminates human error and thus improves precision and sample throughput. In addition, damage to the fragile fiber and consumption of chemicals and their environmental footprint is reduced [64,65]. Automation of dispersive liquid–liquid microextraction based on solidification of floating organic drop (DLLME-SFO) method has been achieved by the integration of a sequential injection analysis (SIA) system with a modified robot that has a 3D printed phase separator [66]. The EME method is automated when performed in a multiwell-format [48]. The HF-LLME method integrates the extraction, purification and concentration steps into a single step and is performed in either a two- or three-phase mode as described in Table 1. Automation of this method is enabled by a 96-well HF-LLME system with an auto-injector integrated to an analytical platform such as high performance liquid-chromatography (HPLC) [51]. Principles of ASE and the factors that determine the efficiency of metabolite extraction are highlighted in [42]. Detailed information on the SFE’s system, procedure, solvents and on-line automation with a supercritical fluid chromatography with a triple quadrupole mass spectrometer detector (SFC-QqQ-MS) are highlighted in the following studies [57,58]. The system and procedure of the MAE method is described by [57,67]. This MAE system has been automated by the incorporation of an autosampler that enables extraction sequences of up to 24 samples and thus accelerates method optimization [59,68]. The principles of the UAE method for metabolite extraction are explained in detail in the studies of [57,69,70].

In the context of plant metabolomics, only a few of these methods have been applied. For instance, the use of SPE to obtain insights into complex phenolic composition of tea and other plant samples [44], SPME to extract metabolites that reflect the changes in the ‘HoneyCrisp’ apples metabolome [71], extraction of phenolics with DLLME for chromatography analysis has been reported by [72], EME with liquid chromatography-tandem mass spectrometry (LC-MS/MS) was used to analyze plant hormones in citrus leaf samples [73], ASE was used in the analysis of natural products in green tea (*Camellia sinensis* L.) [55], SFE had been applied to extract tetrahydrocannabinol (THC) from *Cannabis sativa*L. [74], pharmaceutical and nutraceutical natural compounds from *Berberis* species have been extracted with MAE [75], and UAE has been applied in the extraction of phytochemical compounds from apricot by-products (pulp) [76]. These methods, including HF-LLME and SDME, have not yet been reported in metabolomics studies of plant responses to abiotic stresses. In plant metabolomics, these automated sample preparation methods allow for the extraction of metabolites from hundreds of samples with reduced extraction time and solvent consumption per sample, minimization of variation between samples, improved metabolite recovery, and at higher purity levels. Thus, such automation of sample preparation step enables consistency in sample handling, an assurance factor towards the generation of high quality and reproducible metabolomics data. The confidence in the data produced is essential for maximizing the biological output.

Robotic systems have thus been developed in addition to automated sample preparation methods for rapid throughput and reproducibility in large scale metabolomic analysis (Figure 1). These robots can be incorporated into existing automated systems to function as a transport system (i.e., sample handling) or to perform manipulation (i.e., sample preparation) tasks [77,78]. Depending on the type of task(s) performed, a single-arm or dual-arm robot is used. Single-arm robotic systems perform transportation tasks to connect different stations, whereas dual-arm robotic systems perform both transportation and manipulation tasks with the use of standard laboratory equipment and devices. In addition, dual-arm robots can function simultaneously and independently. Dual-arm robots can also transfer labware from one arm to the other without setting it down [77,79]. An example is the CSDA10F dual-arm robot (Yaskawa, Kitakyushu, Japan), that is used to perform multiple tasks including the handling of vials, solvents, pipettes and sample preparation. It is also capable of transferring and feeding vials to the autosampler of an analytical instrument. The use of the dual-arm robot improved the quality and efficiency of the analytical measurements in comparison to manual sample preparation [79]. Remote sample preparations can thus be achieved with the application of such dual-arm robotic systems, which will save the analyst time and increase reproducibility that will lead to accurate analyses of metabolites. Thus, as one of the core steps in the (plant) metabolomics workflow, sample preparation has a tremendous impact on the final results. Hence, the minimization of errors and improved coverage and reproducibility, by incorporating automation and robotic technologies, will improve the biological insights generated from metabolomics investigations, particularly in abiotic stress studies.

### 2.2. Automation and Analytical Intelligence in Analytical Platforms

Robust analytical platforms are essential for accurate analyses of metabolites with good reproducibility over a period of time. Nuclear magnetic resonance (NMR) and liquid chromatography-mass spectrometry (LC-MS) are the most popular platforms used in metabolomics [29,80,81]. Automation in these analytical platforms, particularly in LC-MS, has significantly improved the efficiency of data acquisition and its reliability. Therefore, due to the preexistence of automation within these platforms, we thus refer to automation in the context of technological advancements of these platforms. In addition, automation has also reduced the risk of variability or errors related to manual operations. However, the operation of these analytical platforms is dependent on highly skilled and experienced analysts that can identify and avoid problems prior to analysis to increase the chance of producing highly reliable and robust data [78,82].

Platforms with analytical intelligence could contribute to the acquisition of highly reliable data, regardless of the user’s skill(s) or experience, but still need highly skilled input to train the algorithms (Figure 1). Analytical intelligence is a concept for analytical instruments and it consists of systems and software that resemble an experienced analyst by automatically identifying good or bad conditions, displaying the results, providing feedback to the user, and solving common problems faced by an experienced analyst. For instance, column equilibration to avoid column damage, constantly monitoring the level and consumption of the mobile phase, management of column performance, manually purging the flow channels and manually picking and integrating peaks (i.e., peak detection and peak integration) are some examples of problems that challenge analyst [78,83,84]. Although different forms of ‘analytical intelligence’ have been part of advancements in analytical systems, the concept of ‘analytical intelligence’ has been recently articulated by Shimadzu [83]. This analytical intelligence means automated support functions that utilize digital technology involving IoT and artificial intelligence to enable high productivity and maximizing reliability, regardless of an operator’s skill level. Analytical intelligence thus allows a system to monitor and diagnose itself, handling any issues during data acquisition without user input, and its implementation is expected to increase data reliability [83].

#### 2.2.1. Mass Spectrometry (MS)-Based Platforms

Automated mass spectrometry (MS) systems are equipped with autosamplers and in some cases cartesian *xyz* robotics systems (i.e., robotics systems designed to permit the arms with at least three degrees of freedom) for sample preparation and injection into LC- and gas chromatography (GC)-MS instruments, thus enabling higher throughput, multiple and remote sample data acquisition [51,78]. GC-MS autosamplers are preferred over the commonly used manual injection procedure, as they provide accurate, precise, and rapid sample aliquoting for large quantities of samples with frequent wash cycles, which reduce sample carryover, defined by the user. Autosamplers thus aid in enhanced statistical analysis and, therefore, reliable biological interpretations by enabling the acquisition and analysis of multiple biological replicates, which increases the sample size for powerful statistical analysis [78,85]. Despite the advances of automated MS systems for enhancing reliable biological interpretations, these systems are, however, limited by their resolving power for isomers. Hence, automated MS systems are coupled with chromatographic separation techniques to improve the separation of isomers. This solution has proven successful; however, for complex samples such as plant extracts, additional advancements are required to drastically improve resolution and thereby enhance metabolite coverage and biological interpretations [34,86].

##### 2.2.1.1. Orthogonal Separations

High peak resolution, metabolite coverage and selectivity can be improved by the development of two-dimensional (2D) GC and LC coupled to MS [34,48]. In these techniques, two columns with different stationary phases, which are connected through a modulator (often involving rotary valves), are used to provide further separation of co-eluting metabolites from the initial column, thus enhancing resolution and peak capacity [34,78]. Considering that plants have complex metabolomes with many unique chemical species and overlapping peaks, the application of these techniques contributes to the advancement of plant metabolomics by enabling the detection, quantification and identification of the vast number of unknown metabolites. Additionally, this will aid in the elucidation of the plants cell and molecular mechanisms [87,88].

Ion mobility spectrometry (IMS) is another technology capable of enhancing peak resolution and selectivity with rapid separations in the millisecond range [89]. IMS devices separate ions based on their differences in mobility under the influence of an electrical field in the gas phase caused by their shape, size and charge. The measurement of the drift time (i.e., mobility) can be converted into the collision cross section (CSS), a unique physiochemical property of an ion [48,90]. IMS is divided into various technologies that are grouped into either dispersive or selective. Dispersive IMS technologies are those that enable the analysis of all ions, whereas selective IMS technologies only enable analysis of selected ions. Hence, the dispersive IMS technologies, which include drift tube IMS (DTIMS) and travelling wave IMS (TWIMS), are suitable for untargeted metabolomics. The selective IMS technologies, which include field asymmetric IMS (FAIMS) and differential mobility analyzers (DMA), are suitable for targeted studies and provide better orthogonality to conventional MS data.

Trapped IMS (TIMS) is the most recent IMS technology that is suited for targeted studies due to its high selectivity with regard to the resolving power of analytes with similar mobility; however, it can be utilized for untargeted studies by separating ions in an ion funnel/drift tube, where the ions are carried by the gas flow towards the funnel exit while the opposed electrical field pushes them back to the entrance funnel, resulting in ions with identical charge to experience the same electrical force but different dragging force from the gas flow due to different CSS. This results in better separation and peak resolution [48,91,92]. The applied field strength, gas pressure and flow in the IMS cell varies among the IMS devices [29,91]. The coupling of IMS with mass spectrometry (IMS-MS) achieves separation based on ion mobility/CSS and mass-to-charge (*m/z*) ratio, which enables separation of isomers and isobars, increases peak capacity, reduces chemical noise and provides structural information through the CCS measurements. IMS-MS thus further enhances resolution, selectivity, and sensitivity [89,91].

IMS can also be integrated into LC-MS systems by incorporating an IMS between the ionization source after LC and before the MS analyzer [93]. For instance, in the study of [94] that resulted in the profiling of 171 metabolites, including phenolics, flavonoids, terpenoids, lipids and nucleotides, in 30 cultivars of leaf and head type lettuces (*Lactuca sativa* L.). An untargeted screening of *Passiflora* leaf extracts with ultra-high performance liquid chromatography ion mobility collision-induced dissociation mass spectrometry (UHPLC-IM-CID-MS) has been used in the identification of flavonoid isomers, i.e., 6-*C* and 8-*C* glycosylflavone isomer pairs orientin/isoorientin and vitexin/isovitexin [95]. These flavonoid isomers have been identified in plants subjected to abiotic stress. Thus, the application of IMS in plant responses to abiotic stress research has the potential to increase the identification of previously unknown detected metabolites and the resultant metabolite coverage, thereby contributing to the elucidation of plant response mechanisms to abiotic stress [96]. IMS is, however, limited by its inability to separate multiple coexisting isomers with similar drift time and biomolecules such as carbohydrates, with flexible structure due to lack of reliable CSS calculation protocols and molecular models. Thus, inaccurate CSS calculations and incorrect structural identification may occur. Here, models based on machine and deep learning algorithms which take into consideration collision effects, the long-range interactions between drift gas and the analyte ion, may be helpful to reduce error rates by computing theoretical CSS values for biomolecules and predicting metabolite structures [97,98]. The characterization of both metabolite structures and spatial information in plants provides the opportunity to elucidate physiological mechanisms in plant organisms, hence the development and advancements of spatial metabolomics techniques [99,100].

##### 2.2.1.2. Spatial Metabolomics: Mass Spectrometry Imaging

Current metabolomic techniques require the extraction of metabolites of interest from biological samples prior to analysis. This approach has been widely used and successful in detecting metabolomic changes in organisms; however, information concerning metabolite spatial location within organelles, cells, tissues or organs is lost in the process, thus making it difficult to interpret metabolomics data. This problem can be resolved but requires time-consuming workflows and expert analyst [101,102]. The development of spatial metabolomics has thus enabled in situ metabolomic approaches.

MS imaging (MSI) is the dominant technology in spatial metabolomics that is used to visualize 2D and 3D spatial distribution of metabolites in biological tissues [102,103]. MSI combines the sensitivity and specificity of MS with the detailed metabolite spatial information, to produce mass spectrums/images representing relative ion intensities at specific tissue localizations [101,102]. MSI platforms are comprised of three components, an ionization source, a mass analyzer and an ion detector. The ionization source extracts and ionizes metabolites simultaneously from the surface of the sample at particular *x*- and *y*-coordinates. The ionized metabolites are separated in the mass analyzer according to their *m/z* and their abundance are detected and recorded by the ion detector. Software is used to produce images, also referred to as intensity maps, from the spatially resolved MS data in which each pixel (i.e., peak) is composed of a mass spectrum. In these images, the spatial coordinates are represented by the *x*- and *y*-coordinates whereas the signal intensity is represented by the color intensity scale [101,104].

**Figure 2:**
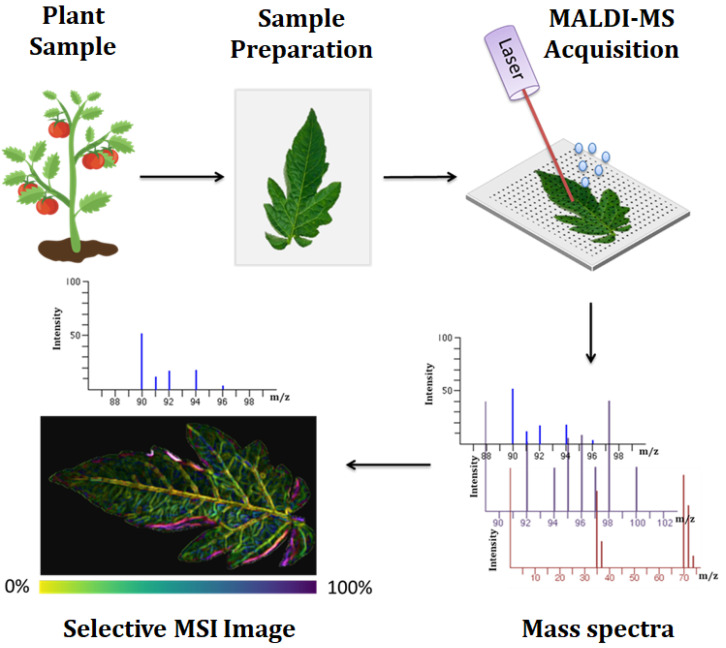
A general overview of a matrix-assisted laser desorption/ionization-mass spectrometry imaging (MALDI-MSI) workflow. A tissue (leaf) is sectioned from the sample (tomato plant) and mounted on a target surface. A matrix is applied to the tissue and a laser beam extracts metabolites into a mass analyzer. Mass spectra are generated at each *x*,*y* coordinates across the tissue surface. A software algorithm combines and processes the mass spectra and generates a MSI image.

MSI platforms are categorized by their ionization source and mass analyzer, and they vary in speed, sensitivity, and spatial resolution. Matrix-assisted laser desorption/ionization (MALDI) is the dominant platform used in MSI and it extracts metabolites and simultaneously ionizes them with the use of a laser (Figure 2). Emergence of other platforms such as desorption electrospray ionization (DESI), laser ablation electrospray ionization (LAESI), nanostructure-initiator MS (NIMS), nanospray desorption electrospray ionization (nanoDESI), infrared matrix-assisted laser desorption electrospray ionization (IR-MALDESI) and secondary ion MS (SIMS), which utilizes an ion beam, increases the development and use of the MSI technology in spatial metabolomics [102,103].

The detection of many metabolites from a single living cell within 10 min, is MSI greatest advantage over current metabolomic techniques. Hence, MSI is increasingly being applied in a variety of biological and clinical studies, as it enables simultaneous measurements of vast amount of metabolites and their spatial distribution from a variety of samples [105,106]. MALDI-MSI has been successful in imaging various classes of endogenous molecules on the surface of different plant organ tissues, including the root, stem, leaf, flower, fruit and seed, to provide new insight into the molecular in situ analysis through the quantitative changes in molecules during plant growth and development or those induced by environmental stresses [100]. The application of MALDI-MSI with on-tissue chemical derivatization of maize root and leaf, has enabled the identification of 600 metabolites [107]. MSI of *Lychnophora salicifolia* leaves with laser desorption ionization (LDI) and MS/MS, revealed the accumulation of vicenin-2, a di-*C*-glycosyl flavonoid, in the mesophyll cells and upper epidermis, in response to extreme sunlight [108]. Spatial metabolomics has also been used to explore plant specialized metabolite diversity in the plant genus *Euphorbia L.*, and the diterpenoids were shown to be localized to young stems and roots, thereby suggesting they play a role in defense responses to biotic stresses [109].

The widespread application of spatial metabolomics is likely to benefit from the incorporation of machine and deep learning techniques, as well as cloud computing. For instance, the development of novel mass spectrometry imaging software, in addition to several existing examples of software such as MSiReader [110], with ML capabilities to predict mass accuracy of collected data, will enable reduction in molecular ambiguity, enhanced data quality and interpretability. Machine learning dimension reduction methods, such as *t*-distributed stochastic neighbor embedding (t-SNE) and uniform manifold approximation and projection (UMAP), enable visualization of the large complex image spectral data that will aid in identifying similar pixel or image clusters and data interpretation [102,111]. Cloud computing platforms such as METASPACE and OpenMSI, have enabled the construction of imaging MS libraries that contribute to rapid and accurate metabolite identification by resolving ionization pathways and integrating all signals corresponding to a particular metabolite, and data analysis, thus increasing the widespread use of MSI [102]. The application of spatial metabolomics thus has the potential to further elucidate plant response mechanisms to abiotic stresses, as MSI enables rapid metabolite detection and identification, particularly of those lost or degraded in the extraction process, and the discovery of novel metabolites, that will provide insights of the metabolome changes in response to the abiotic stresses, within the organelles, cells, tissues or organs. Additional technologies for high throughput and automated analysis have been developed.

##### 2.2.1.3. Lab-On-Chip and Microfluidic Devices

The emergence of microfluidic or lab-on-chip technology enables the integration of multiple analytical processes onto a single platform. The technology is based on the fabrication of microdevices with small channels and chambers that enables the manipulation of fluid volumes in the micrometer range and parallel workflows for automated and high throughput analysis [112,113]. The integrated processes in microfluidic devices include sample preparation, preconcentration, separation and delivery to analytical platforms [48,112]. Microfluidic platforms are divided into three categories: analog, droplet and digital microfluidics (DMF). Analog and droplet based microfluidic platforms utilize fluid shear stress between an oil phase and an aqueous phase, to generate continuous fluid streams and separate droplets within enclosed microchannels, using passive and active pumping mechanisms [114,115]. DMF platforms utilize electrodes, coated with a hydrophobic material layer, to move, mix, merge and separate samples when a voltage potential is applied [112,114]. The handling of small fluid volumes with low flow rates make microfluidic devices suitable for coupling with MS. Soft ionization techniques are usually employed for the ionization of delicate biomolecules, molecules that are sensitive to heat and are usually large polar organic molecules [78,116].

The coupling of microfluidic devices with electrospray ionization-mass spectrometry (ESI-MS) is dependent on emitters that are either integrated or external to the device [112,114]. Description of the emitters are well documented in [112]. MS analysis in droplet-based microfluidic platforms is performed by either extracting the droplets from the multiphase flow or by directly delivery of droplets to the MS without extraction. The coupling of DMF devices to ESI-MS requires the transfer of droplets from the device to the ESI emitters and the dissociation of the droplet manipulation and spray voltages. This is achieved by using sandwiched capillary emitters and pump based sampling capillaries connected to external emitters for closed and open systems, respectively [112,114]. Generally microfluidic platforms are coupled offline with MALDI-MS by either spotting samples onto MALDI plates or by direct MALDI analysis of the fabricated material of the microfluidic device. Online coupling has, however, been established [78,114].

Analog platforms utilize droplet ejectors such as embedded capillaries or integrated piezoelectric microdispenser for target spotting and they can be programmed for automated positioning. Droplet and DMF platforms can be coupled online as they enable contactless spotting of samples by exploiting their segmented flow. The precise handling of small fluids and the automation of sample preparation, concentration and separation provided by microfluidic platforms has improved MS sensitivity and reproducibility. Microfluidics can also increase separation times as the distance or steps between the separation and MS detector is reduced [48,114]. The microfluidic device, RootChip, has been applied for measuring changes in metabolite concentrations, such as calcium and phytohormones, and revealing the specific signal transduction responses in *Arabidopsis thaliana* roots treated with biotic (flg22) and abiotic (sodium chloride (NaCl)) elicitors [117]. The RootChip allows the simultaneous stimulation of a single organ; in this case, the treatments were applied on one side of the root, while the cell responses are studied on the untreated side. The RootChip device can thus enable the testing of combined treatments without exposing the cells to the individual treatments. Microfluidics in plant–environment interaction studies can, therefore, enable the elucidation of environmental stress sensing, signaling, and intercellular and molecular mechanisms [117]. Microfluidics can deliver fluid droplets directly into MS analytical systems to increase throughput analysis and data reliability. The improvement of data reliability and accurate mass detection through the development of novel data acquisition workflows/algorithms is an active research area.

##### 2.2.1.4. Virtual Metabolomics Mass Spectrometer

Emerging analytical workflows for MS and MS/MS spectral data acquisition are bound to improve accurate mass detection and thereby enhance metabolite identification. The coupling of chromatographic separation with MS/MS is the widely used and one of the most powerful approaches used for metabolite identification. MS/MS spectra are obtained by either data-dependent acquisition (DDA) or data-independent acquisition (DIA). In the DDA workflow, the MS instrument changes from a full scan MS to MS/MS when precursor ions overcome a predefined threshold of intensity or other criteria such as isotope pattern, mass defect, and the presence of diagnostic ion or characteristic neutral loss, thus resulting in poorer sampling of lower abundant precursor ions. DIA workflows, such as MS^E^, involve alternating scans acquired at low or high collision energy to obtain full scan precursor ions, accurate mass fragments and neutral loss information. However, matching the precursor ions to product ions when there is substantial chromatographic co-elution or overlap is challenging. Algorithms are thus employed to intelligently select precursor ions, thus improving the efficiency of MS/MS spectral acquisition [29,118,119].

Recent software such as Virtual Metabolomics Mass Spectrometer (ViMMS) enables the prototyping, implementation, optimization and validation of new acquisition methods in silico [119,120]. These algorithms improve MS/MS acquisition by determining precursor cleanliness or by reducing irrelevant spectra acquisition and automatically adjusting to various chromatographic conditions to provide improved reproducibility [118,121]. The intelligent automation of precursor ion selection reduces the need for highly experienced or skilled analyst and makes the successive metabolite identification process less challenging [78,121]. Such an efficiency gain could also spark a renewed interest in LC-MS*^n^*-based approaches where spectral trees are formed to gain deeper insight in the fragmentation pathways involved in the breaking apart of the metabolite structures, thereby facilitating metabolite annotation as shown for polyphenols standards and flavonoids in plant extracts [122,123]. In addition to the aforementioned technologies in MS-based platforms, advances in NMR for improved metabolite resolution and identification have been made [124].

#### 2.2.2. Nuclear Magnetic Resonance (NMR)-Based Platforms

NMR is popular for its high reproducibility; however, in contrast to MS, it has poorer sensitivity, lower dynamic range and resolving power and, therefore, it typically results in a restricted metabolite coverage in plant metabolomic studies. NMR technology has recently been improved by the development of miniaturized radiofrequency coils, superconducting magnets, cryogenic probes and multidimensional NMR techniques [18,125]. These developments have thus enabled the improvement of resolution, acquisition time, multi-nuclei detection and performance of magnets at temperatures close to 4 Kelvin (K), due to using cryocoolers or closed cycle helium (He) refrigerators that automatically recycle He, thus eliminating the need for constant liquid helium (He) or liquid nitrogen (N_2_) refills [125,126]. The study of metabolites by NMR was enabled by the ^13^C optimized cryogenic probes. The development of a ^1^H-^13^C dual-optimized probes enables 2D NMR experiments at natural abundance [125].

The application of 2D NMR improves NMR sensitivity by reducing resonance overlap and provides information on chemical bonding between nuclei. However, 2D NMR experiments are time consuming and therefore challenging to use in large-scale metabolomic studies [29]. Acquisition time in 2D NMR experiments can be increased by various approaches. One approach is the use of diffusion and/or relaxation filters that reduce the interscan delay (i.e., relaxation time). Non-uniform sampling (NUS) is another approach that entails the recording of randomized data points instead of all data points. Ultrafast (UF) 2D NMR is another approach that enables the acquisition of a 2D spectrum in a single scan. These approaches are described in more detail in [34,125]. Metabolite fingerprinting of *Bougainvillea spectabilis* leaves exposed to high levels of air pollution was achieved with 2D NMR, and revealed the differential concentrations of amino acids, sugars, krebs cycle intermediates, phenylpropanoids, flavonoids, and production of putrescine, gamma-aminobutyric acid (GABA) and trigonelline metabolites in response to the air pollution stress [127]. Additional technologies have also been developed to enhance NMR sensitivity of which some are highlighted in the next paragraphs.

Dynamic nuclear polarization (DNP) is another advancement that enhances NMR sensitivity. DNP enhances the NMR signal by transferring the polarization of the electron spins to the nuclei prior to delivering the sample to the NMR spectrometer. DNP thus allows the detection of metabolites, particularly natural products, present in low concentration in complex mixtures [34,126]. DNP has been applied for the abundant detection of ^13^C nuclei in the metabolomic analysis of tomato extracts [128]. DNP has the potential of increasing metabolite coverage to facilitate the use of NMR in untargeted studies of plant responses to environmental conditions.

Additionally, the detection of metabolites within intact tissue samples has been made possible by the advancements in high-resolution magic-angle spinning NMR spectroscopy (HRMAS) [126]. The development of microprobes for magic-angle spinning (µMAS) of sub-microgram specimens with high-resolution in HRMAS, allows site-specific metabolomic characterization of varying plant and tissue regions without the need for sample preparation. Therefore, HRMAS provides the opportunity to explore the specific tissue metabolism with more or less the same precision as MSI [126]. HRMAS has been applied to monitor plant responses to abiotic stresses. For instance, the study of soybean leaves grown with and without water-deficiency stress with HRMAS, revealed differences in the metabolite profiles. A total of 30 metabolites were identified, with the amino acid metabolites present in the metabolite profile of soybean leaves with water-deficient stress [129]. HRMAS can therefore aid in elucidating plant response mechanisms. The identification of novel metabolites will greatly contribute to the discovery of unidentified pathways and thus the elucidation of plant response mechanisms.

The de novo identification of novel metabolites from complex mixtures with NMR can be enhanced with on-line hyphenation of separation techniques. A flow cell matching the high performance liquid-chromatography (HPLC) module is used to connect the NMR, instead of a probe, thus resulting in HPLC-NMR. In HPLC-NMR, the separated analyte is eluted from the column to the flow cell and subsequently to the NMR spectrophotometer [34]. High performance liquid-chromatography-solid phase extraction-NMR (HPLC-SPE-NMR) is another hyphenated NMR technique that enriches the analyte by allowing the removal of the HPLC mobile phase, prior to NMR data acquisition. HPLC-SPE-NMR decreases the analyte chromatographic peak volume and, therefore, achieves greater sensitivity compared to HPLC-NMR. Additionally, HPLC-SPE-NMR has been enhanced with the integration of a photodiode array detector (PDA) and MS for authentication and the structural characterization of secondary metabolites from complex plant extracts [34,130]. For instance, HPLC-PDA-HRMS-SPE-NMR analysis of *Coleonema album* leaves enabled the structural identification of 23 coumarins [131]. An automated HPLC-MS-SPE-NMR approach was used to characterize flavonoid structures in crude tomato extracts [130]. Despite the accomplishments of these technologies in improving NMR sensitivity, resolution and acquisition time, advances in software tools and databases are still required to aid in metabolite analysis [125].

Novel software tools consisting of machine and deep learning algorithms have been developed to provide accurate prediction of chemical shifts and automation to manual phasing, water removal, baseline correction, peak picking and spectral fitting methods, thereby improving accurate quantification of metabolites for reliable structural elucidation. These automatic deconvolution algorithms include Bayesian Automated Metabolite Analyser for NMR spectra (BATMAN) [132], Bayesil [125], AQuA [133], MagMet [125] and rDolphin [134], and they contribute to making NMR faster, consistent and user-friendly. The integration of this software with online websites/databases such as NMRShiftDB further enhances NMR accuracy and molecular coverage with increasing data, thus enhancing structural discoveries and elucidation [84,125,135]. Hence, recent efforts have been made to make NMR data publicly available through the development of software tools and databases for the discovery and structural elucidation of natural products [136].

Small Molecules Accurate Recognition Technology (SMART), a machine learning based tool, for example, enables the interpretation of 2D-NMR spectra and the acceleration of discovering and characterizing novel natural products [137]. Additional examples include the ImatraNMR [138] and SimpeleNMR [138] tools for quantitative NMR analysis. ImatraNMR and SimpeleNMR tools were applied in the 2D NMR analysis of 4 *Arabidopsis thaliana* strains under controlled and ozone exposure conditions. Metabolite differences were revealed, with GABA found to be produced in the plants responses to the ozone exposure (i.e., changes in oxidative stress) [138]. These 4IR technological and software developments in NMR will, therefore, enable rapid analysis for large datasets and potentially increase the widespread use of NMR-based plant metabolomics [139]. In this review, we mainly focus on LC-MS and LC-MS/MS platforms and their data processing and analysis workflows.

### 2.3. Machine Learning Methods for Metabolomic Data Mining and Interpretation

**Figure 3:**
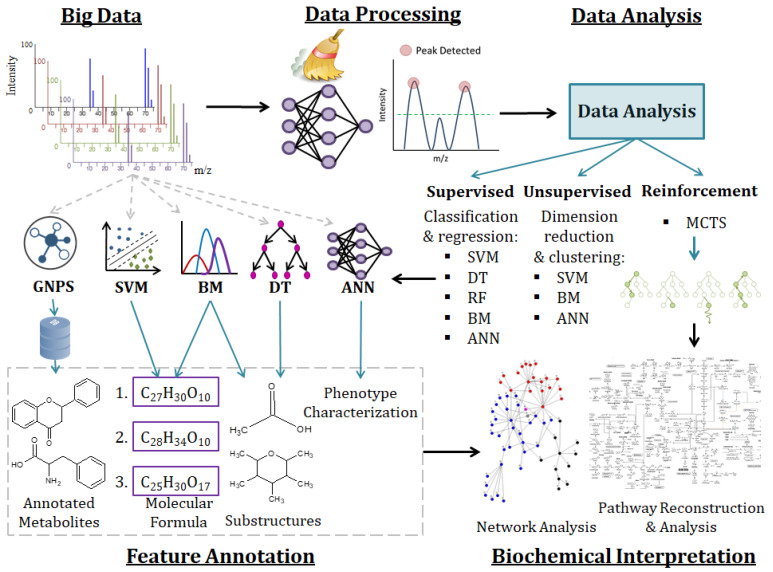
Workflow for metabolomics data analysis and biochemical interpretation with machine and deep learning algorithms. Various machine and deep learning algorithms are implemented all through the analysis and interpretation workflow. Artificial neural networks (ANNs) implemented in data processing remove noise and artifacts from raw data by performing peak detection and alignment. In data analysis, ANNs, support vector machines (SVMs) and Bayesian models (BMs) may be applied as either unsupervised or supervised methods and, in the former, they explore and reduce the dimensionality of the data. The supervised analysis with SVMs, decision trees (DTs), random forest (RF), BMs and ANNs, classify and extracts/selects relevant features from the dataset for feature annotation. Selected features from data analysis are used as input data for GNPS, SVMs, BMs, DTs and ANNs. Raw spectral data may also be used as input data. GNPS queries the data against its spectral library to annotate the selected features. SVMs and BMs predict and rank the features molecular formula. Additional BMs and DTs extract substructures from the data. ANNs characterize phenotypes from the data. The annotated features are integrated and applied for network analysis, pathway reconstruction and analysis. Abbreviations: ANN, Artificial neural network; SVM, Support vector machine; BM, Bayesian model; DT, Decision tree; RF, Random forest; MCTS, Monte Carlo tree search; GNPS, Global Natural Product Molecular Networking.

Axiomatically, metabolomics studies, particularly untargeted approaches, generate complex, with inherent covariance structure, and information-rich big data sets that are challenging to handle and interrogate. With technological advancements in analytical platforms (Section 2.2) and an increasing complexity in the study design, untargeted metabolomics data are increasingly becoming more heterogeneous, big data in terms of volume, velocity and variety [5,140]. Thus, the availability of big data in (plant) metabolomics reflects a new era of data-driven research employing powerful computational tools, involving AI and machine learning algorithms, to maximally mine and find novel knowledge in this (big) data [5,141]. The typical approach used in mining and interpreting untargeted metabolomics data is well-described in literature; it is a multistep workflow, employing dedicated mathematical modeling and chemometric algorithms (Figure 3) [26,142,143].

The philosophies of data science—the extracting of information from (big) data—have recently undergone an *aggiornamento*, with a resurgence in interests in linear and nonlinear machine learning (ML) methods [144,145,146]. Furthermore, historically, nonlinear ML methods have not been widely used due to difficulty in deriving statistical inference, and thus biological interpretation. However, with increasing appreciation of nonlinearity in biological (big) data, nonlinear ML algorithms are increasingly being explored and applied in interrogating data, in life sciences [102,147,148,149,150]. ML is a subdomain of AI that provides machines with the ability to learn directly from data and past experiences through computational algorithms to facilitate better informed decisions or actions, without the involvement of experts. ML methods are categorized as either supervised-, unsupervised-, or reinforcement learning algorithms [151]. The algorithms can be further categorized based on their learning techniques, which are classification, regression, clustering, and dimensionality reduction (Figure 3) [151,152].

In plant metabolomics, several ML-based methods (Figure 3) are increasingly being explored to attain a comprehensive insight and understanding of the plant biological systems under different conditions (Table 2). Deep learning (DL) algorithms and artificial neural networks (ANNs) have been employed in biomarker discovery from natural products/herbal medicines [153,154]. Additionally, [155] explored ANN and DL algorithms in their studies to reveal the diversity of specialised metabolome in different *Camelina sativa* varieties. Deep convolutional neural networks (DCNN) have also been used as an alternative of ANNs in the mass spectrometry imaging-based detection of specific secondary metabolites in tomato [156]. Support vector machine (SVM) has been applied in plant metabolomics research for the classification, identification and predictive power of metabolite content in exploring the medicinal properties of plants [148,157]. Random forests (RF) have also been used in optimization of metabolic fingerprinting and metabolite detection studies [158,159]. Other ML-based methods such as the nearest neighbour, Naive Bayes and decision trees have also been used in biological pathway predictions for a deeper understanding of metabolic pathways and networks in plants for crop improvement [160,161].

However, in metabolomics studies that investigate plant responses to abiotic stresses, the exploration and application of ML methods is still to be encouraged. The integration and use of these ML and deep learning (DL) algorithms in the metabolomics data analysis pipeline hold the promise of transforming the future of metabolomics research. The ML and DL methods offer the flexibility to effectively analyze and integrate a large volume of multi-omics data (e.g., metabolomics, transcriptomics, genomics, and proteomics) given a large enough sample size and enough biological context [162,163], thus providing better predictive models. This would advance the understanding of regulatory network rules and mechanistic events at cellular and chemical space of the plant, illuminating its responses to abiotic stresses. Thus, the paragraphs below dive into describing some of the ML methods, pointing out their applications in metabolomics studies to illustrate their potentials to support metabolomics analysis workflows and facilitate possible integration with other omics data types.

#### 2.3.1. Support Vector Machines

Support vector machines (SVMs) are classification and regression ML algorithms that separate data into two classes [165,166]. SVM algorithms map samples as data points from various classes in a high-dimensional feature space. The SVM then constructs an optimal hyperplane that maximizes the distance to the nearest point of each class (i.e., margin) [146,167]. Maximizing the margin enables the SVMs to correctly classify new data points that lie within the margin on either side of the hyperplane [146]. SVMs have been reported to outperform PLS-discriminant analysis (PLS-DA) for feature extraction and classification accuracy of metabolomics data [168].

The support vector machine recursive feature elimination (SVM-RFE) algorithm has proven useful in identifying metabolic biomarkers in untargeted LC-MS datasets. This iterative algorithm ranks metabolites according to their ability in discriminating the two phenotypes/conditions (i.e., control and treated samples). Features most informative of discriminating phenotype or condition are ranked higher than those less informative, thus this ranking system is ideal for biomarker discovery as it ranks based on predictive accuracy [167]. The clustered support vector machine (CSVM), a clustering ML algorithm, divides input data into multiple clusters and trains a SVM within each cluster to separate the data in the clustered feature space, thus reducing the number of relevant features [169,170]. Furthermore, SVMs also play a role in metabolite annotation workflows, as in the SIRIUS 4, a tool for molecular structure identification, an SVM is used to aid in the determination of a molecular formula for a candidate molecule (Figure 3) [171].

SVMs can thus facilitate the visualization of relationships between plant samples, their classification and discovery of contributing markers or natural products that correlate to biological origin, such as abiotic stress, with strong confidence [148,172]. SVMs, as with the classical supervised statistical methods, are, however, widely used for the discrimination of two different classes and, due to their limitations, they are rarely used for multi-class analysis. Hence, ML algorithms such as decision trees (DTs) have been developed and applied for the analysis of plant complex multifactorial characteristics [167,173,174].

#### 2.3.2. Decision Trees

Decision trees (DTs) are ML algorithms that perform classification and/or regression of both categorical and continuous input and output variables in a tree-like structure composed of a root node, internal nodes and leaf nodes. A DT organizes or separates dataset (i.e., input at the root node) into smaller homogenous subsets or sub-populations (i.e., output at the leaf nodes) based on the most significant classifier among the independent variables [175,176]. The internal nodes represent the values of the attributes, whereas the leaf nodes represent the final decisions or predictions and the label of the class after following the path from the root to the leaf nodes [166].

In plant metabolomics, DTs have been applied in the extraction of metabolite features that discriminate different rice cultivars [173], in the identification of 11 anti-inflammatory biomarkers from 57 *Asteraceae* species leaf extracts [153] and in the prediction of the most frequent substructures based on the mass spectral features and retention index (Figure 3) [177]. A DT based on the fragmentation patterns of metabolites approach was used for the annotation of 28 unique avenanthramides in oat seedling extracts [178]. Additionally, DTs have also been used in predicting plant responses to drought, salinity, heat and cold stress [174]. DTs are, however, underexplored in plant metabolomics and their applications could provide the opportunity for multifactorial plant characteristic analysis, such as the investigation of plant response mechanisms to multiple stresses, and identification of discriminant metabolite biomarkers that correlate to the abiotic stress within the complex metabolomics data [179]. A general advantage of DTs is their interpretability as compared to many other classifiers such as random forest (RF) discussed in the next section. However, the comprehensibility of DTs is at the expense of lower predictive accuracy compared to other classifiers such as SVMs. Fortunately, the classification accuracy of DTs can be improved by ensemble learning (EL) methods [180].

#### 2.3.3. Ensemble Learning

Ensemble learning (EL) methods are used to enhance the predictive performance of statistical learning and model fitting techniques by constructing a linear combination of a base learning algorithm. Common EL methods include random forest (RF) and bootstrap aggregating or bagging algorithms [175,176]. RF is a decision tree-based classification algorithm that is capable of handling high-dimensional data with many features, noise, unbalanced and missing values. RF aggregates multiple decision trees through bagging and bootstrapping methods and classifies new data based on the consensus of the classification trees [166,181]. RF, unlike DT algorithms, separates a sample of features for each separation instead of trying to separate all the features, thus reducing the variance and improve predictive accuracy. Bagging is an approach that repeatedly extracts samples from the same set of data, and with the use of bootstrapping methods, the predictability of this approach for classification is maximized through the direct log-likelihood optimization [166,176]. RFs, in addition to SVMs, are the widely applied ML algorithms in life sciences [182].

In omics sciences, RF has aided in extracting biological insights by detecting genotype from environment interactions [183]. RFs are, however, relatively new to metabolomics studies and are proving to be powerful classifiers due to their high classification accuracy, ability to determine the variable/feature importance (i.e., identifying variables that contribute to the prediction results) (Figure 3), avoidance of overfitting and tolerance to outliers and missing values [184,185,186]. Additionally, RFs have been reported to perform better on metabolomics data for phenotypic classification and feature extraction, as compared to other classification methods. For instance, RF with 100% accuracy outperformed the SVM, PLS and linear discriminant analysis (LDA) classifiers in the analysis of clinical metabolomic data of healthy and colorectal cancer diagnosed patients’ urinary samples [185,187].

Considering clinical metabolomic data are relatively similar, in terms of complexity and size, to plant metabolomic data, the application of RFs in plant metabolomics, particularly for plant-environmental data, thus offers great potential to accurately extract features that discriminate the plant phenotypic responses to environmental conditions such as abiotic stresses, and therefore improving data interpretation. RFs are, however, disadvantaged by their complex visualization and lack of statistical significance measurements such as a p-value. RFs thus provide a list of the most important metabolites without a cut-off value to select any “significant metabolites”. The feature extraction process for RFs is, however, not reliable in situations where variables differ in their scale of measurement or number of categories [168,188]. Hence, alternative approaches, such as Bayesian models (BMs), with enhanced feature extraction and high reproducibility of metabolomic data, have been considered for such situations [189,190].

#### 2.3.4. Bayesian Models

Bayesian models (BMs) are a group of probabilistic models that conduct their analyses based on the Bayes’ theorem, which calculates the probability using the previously obtained probability and information of the data collected. BMs are employed for either classification or regression problems and clustering [175,176,191]. BMs modify probability distribution to identify possible concepts by assuming independence between the variables and calculating the conditional probability for each instance based on the assumed classes [166,176]. BMs are promising for metabolomic analysis as they allow the incorporation of prior knowledge with experimental data to facilitate better predictions, capture relationships in non-linear interactions between metabolites and phenotypes, reduce model overfitting, and identify significant individual metabolites [189,190].

BMs applied in metabolomics have resulted in confident/reliable compound annotations through Bayesian-based tools such as the Integrated Probabilistic Annotation (IPA) method. The IPA method incorporates multiple sources of information within the annotation process to increase the predictive power of assigning measured *m/z* values to putative formulas (Figure 3). Additionally, the confidence in annotations are quantified and re-evaluated when new information is provided, thus improving annotations, particularly for data obtained from similar biological samples using the same experimental procedure [192]. MS2LDA, a Bayesian Latent Dirichlet Allocation (LDA) model, extracts peak patterns in LC-MS/MS data that represent molecular substructures (Figure 3) to enable the grouping of molecules based on shared substructures regardless of overall spectral similarity, thus enhancing the extraction of plant metabolite building blocks that facilitate metabolite annotation and that could be linked to phenotypic differences through differential analysis [8]. SIRIUS 4.0, a computational tool for metabolite identification from MS/MS data, implements a Bayesian network scoring that enables it to identify the molecular formula and structure of a query compound with high accuracy (Figure 3), thus resulting in enhanced and more confident compound identification [171,193].

The identification of molecular formulas and structures for large compounds (i.e., >500 Da) is, however, still challenging, particularly for plant extracts that contain large conjugated compounds above 700 Da. Organic compound Determination by Integral Assignment of elemental Compositions (ZODIAC), a network-based algorithm for estimating molecular formula, is an alternative approach that combats this problem. ZODIAC uses Bayesian statistics to re-rank SIRIUS molecular formula candidates. The ZODIAC score increases the confidence in formula annotation, particularly for large compounds, and reduces the error rates. Analysis of tomato (*Solanum lycopersicum*) extracts with ZODIAC resulted in the decrease in error rates from 4.44% to 2.22% and the discovery of three novel molecular formulas not found in any structural databases [194]. These powerful Bayesian-based tools can, therefore, contribute to the elucidation of plant response mechanisms to abiotic stress by facilitating the accurate extraction of structural properties and identification of novel metabolites involved in the plants response to abiotic stress. BMs can, however, be complex, difficult to implement, and computationally quite expensive, in terms of the power requirements. Hence, alternative methods are also considered such as artificial neural networks (ANNs) [195,196].

#### 2.3.5. Artificial Neural Networks

Despite the considerable efforts of the aforementioned ML methods to rapidly and accurately characterize non-linear complex samples and their metabolite properties, false positive signals, co-eluting metabolites and retention time shifts are still major bottlenecks that effect data analysis and interpretation of plant MS-based metabolomics. Artificial neural networks (ANNs) and deep learning (DL) methods are proposed to solve these issues and other bottlenecks involved in the mining of metabolomics data [154,197].

ANNs are interconnected information processing systems that resemble the human’s nervous system. ANNs can be used for classification and/or regression of non-linear systems and are characterized by their architecture, patterns of interconnected processing units (i.e., neurons), method of determining the weights on the connections (i.e., learning algorithm) and their activation function [176,198]. The general ANN architecture consists of an input layer, where the data is introduced into the system, hidden layer(s), where learning occurs and the information obtained is linked to the output layer that provides the resultant decision/prediction. The connections are based on the weight values defined during the training process and, therefore, the output values will be very close to those defined in the training model [166,175]. ANNs can be divided into two categories: conventional ANNs and deep ANNs, also referred to as DL or deep neural networks (DNNs). DNNs have multiple processing layers (i.e., multiple hidden layers) that enable them to learn and fit raw data through representation at multiple levels of hidden layers. Hence, more and improved representation of observed patterns in the upper or upstream layers is achieved [162,175]. DNNs are advantageous in their ability to extract features automatically, thus eliminating potential bias, and to function as either a supervised or unsupervised method. The commonly used DNN is the convolution neural network (CNN). Alternative DNNs include unsupervised pretrained networks, recurrent neural network (RNN) and recursive neural network [175,197,199]. DNN and ANN models have been widely applied in the data processing and data interpretation steps of metabolomic pipelines (Figure 3) [197,200].

An optimized back-propagation ANN (BP-ANN) was applied in the classification and discovery of quality markers with bioactivity from the Jinqi Jiangtang (JQJT), a Chinese herbal medicine, metabolomic dataset. The BP-ANN outperformed the PLS in terms of accuracy and low error rate [154]. DNNs reported in metabolomics studies include a CNN model, peakonly [201], for peak detection and integration. The peakonly algorithm classifies LC-MS data into regions of noise, chemical peaks and uncertain peaks, thereby eliminating false noise peaks and determines peak boundaries for integration. DeepSpectra [202], a CNN-based model, enables phenotype characterization directly from raw spectral data. DeepSpectra has been applied in the prediction of biomass and protein content in environmental datasets. ChromAlignNet, a RNN-based model, for peak alignment of GC-MS data enables the alignment of complex data without the requirement of additional parameter selection and reference chromatograms [197,200]. Furthermore, DNNs have also been used to predict collision cross section (CSS) values, properties of ion-mobility MS that can be used to eliminate the time-consuming search of unknown metabolite identification by matching the CSS values with metabolites in spectral databases, which will also increase confidence in identification [197]. ANNs are showing great promise in detecting plant diseases and discriminating between healthy and diseased plants through hyperspectral imaging data. The advancement of these models in hyperspectral imaging could translate to the development of ANNs for mass spectrometry imaging (MSI) [203].

Altogether, plant metabolomic studies are likely to benefit from such versatile ANN and DL models, due to their ability to encode and model complex non-linear datasets. For instance, in the case of investigating plant response mechanisms to abiotic stresses, ANN and DL models will facilitate classification and extraction of relevant features with high accuracy from raw data, due to their ability to eliminate noise and false signals, and separate co-eluting metabolites. Additionally, their capability in predicting the presence/absence of substructures with high accuracy based on the spectral information and molecular formulas, will facilitate enhanced metabolite identification and discovery of novel metabolites that will aid in elucidating plant response mechanisms to abiotic stress and the modeling of active pathways [197].

#### 2.3.6. Machine Learning for Pathway Modeling

The identification and understanding of metabolic pathways is important in elucidating the underlying biological processes/mechanisms, such as the causes of metabolite diversity, of an organism. Substantial knowledge on metabolic pathways, particularly plant pathways, have been elucidated and curated in various databases such as Kyoto Gene and Genome Encyclopedia (KEGG); however, there still remain unidentified metabolic pathways with their associated gene–enzyme–metabolite relationships [204,205]. The reconstruction of metabolic pathways assists in the identification of unidentified pathways. This process is complex and widely based on hypothesis-driven models (i.e., constraint-based and kinetic models) that use gene annotation and ontology, prerequisite knowledge that include stoichiometry, thermodynamic and evolutionary assumptions, and enzyme kinetics [161,206,207].

The reconstruction of metabolic pathways is, however, challenged by the vast number of genes belonging to large gene families, which encode metabolic enzymes, and the expensive, time-consuming biochemical and genetic experimental approaches [205,208]. Additionally, the advancements of data acquisition technologies have resulted in increased large and complex omics datasets, which makes it challenging for hypothesis-driven models to incorporate and extract meaningful information from the data for pathway modeling [206,209]. For instance, constraint-based models (CBMs) are limited by their steady-state assumption and insufficiency to accurately capture cellular dynamics such as metabolic responses, whereas kinetic models are limited by the unknown mechanistic kinetic rate law for most enzymes involved in specific reactions and the computational challenges associated with parameter estimation and model expansion [207,210,211]. Hence, alternative mathematical and computational models with the capability of predicting organism-specific metabolic pathways directly from omics data are required to address these challenges. Data-driven models have been reported to predict biological systems’ behavior, without prerequisite knowledge, from captured data [206,207,212].

Data-driven models are based on ML principles that enable them to predict pathway dynamics from experimental data without any knowledge on the organism’s metabolism. This systematic approach increases the prediction accuracy with increasing data [210]. However, data-driven models cannot provide mechanistic explanations of phenomena, thus they are combined with hypothesis-driven models to provide mechanistic insights in a sample-specific manner [209,211]. In such hybrid-models, experimental data is integrated with the hypothesis-driven models to extract mechanistic information that is provided as input data for unsupervised or supervised ML algorithms that result in output data as relevant features that improve prediction accuracy and genotype to phenotype predictions [36,213]. ML algorithms optimize CBMs and kinetic models using known pathway instances curated in databases as training data to facilitate predictions based on experimental data and not exclusively on prior knowledge [207], to extract features in existing pathway and models [36], and to map multi-omic data onto a metabolic model that contains different integrated omics and model data through different ML algorithms [214]. Furthermore, the integration of ML algorithms with hypothesis-driven models, enables the models to accurately extract complex patterns from non-linear biological samples, improve predictability and provide cause–effect information about the metabolites in the pathway [209,214,215]. Several tools have been developed based on the aforementioned ML algorithms with the aim of modeling metabolic pathways.

Probabilistic modeling for Untargeted Metabolomics Analysis (PUMA), a Bayesian inference-based probabilistic approach, is an example of a model that predicts pathways that are likely to be active based on the metabolomics measurements, followed by probabilistic annotations, which assign chemical identities to the measurements. A pathway is predicted to be active if the measurements generated from a particular path is greater than the user-defined threshold [216]. The recently developed Lilikoi v2.0 R software, implements a DL neural network classifier in addition to six ML algorithms including RF and SVM, for the prediction and visualization of metabolic pathways. Lilikoi requires metabolomics data matrix with a column of categorial variables (i.e., treated/control) for classification as input data, which is analyzed prior to the construction of the pathway [217]. RetroPath Reinforcement Learning-based metabolic space exploration (RetroPath RL) implements the Monte Carlo Tree Search (MCTS) reinforcement learning method (Figure 3) to discover and suggest experimentally relevant pathways. Due to its focus on exploring the research space, RetroPath RL can propose multiple pathways for the same compound. This feature will greatly aid in the elucidation of plant response mechanisms to stresses, as plants consist of metabolites involved in multiple pathways. RetroPath RL, however, has its limits, particularly in pathway ranking [218,219].

Ranking pathways with accuracy has been achieved by the adoption of a SVM in the multi-label classifier, iMPT-FRAKEL, which uses compound fingerprints to identify metabolic pathways of compounds [204]. Pathway Activity Level Scoring (PALS), available as a Python library, command line tool, and web application, analyzes metabolites grouped as metabolic pathways by ranking changing pathways or intensities in sets of metabolites over different experimental conditions [220]. The aforementioned ML-based tools or models hold great potential in the identification and discovery of pathways from poorly or lack of characterized experimental data and highlight information on signaling pathways, gene expression regulation and epigenetics within the system studied. The extraction of such information in plant-environmental metabolomic studies will contribute to the elucidation of plant response mechanisms to stress. Furthermore, the differences in plant response mechanisms to specific stresses can also be highlighted.

### 2.4. Large-Scale Metabolite Annotation

**Figure 4:**
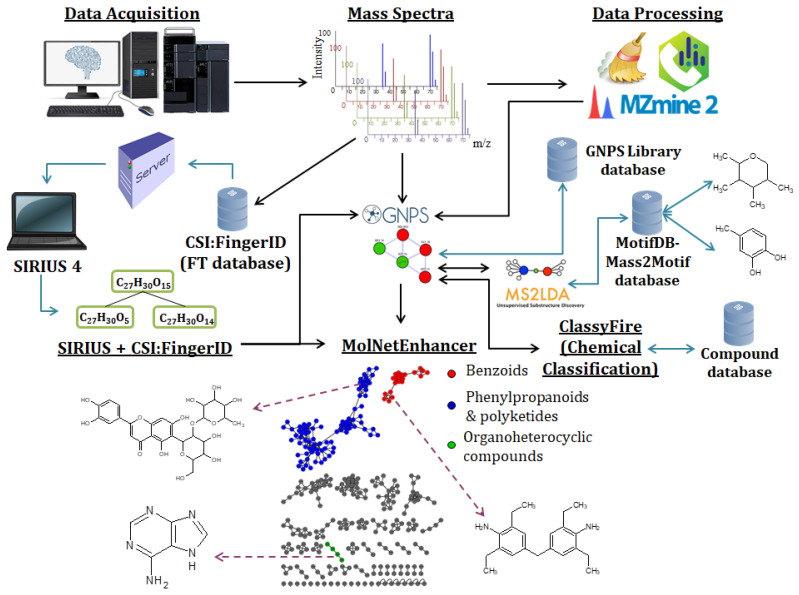
A schematic overview of 4IR technologies applied in metabolomics for large-scale automatic metabolite annotation and exploration by molecular networking. 4IR technologies include analytical intelligence (AI) that ensures high reliable/quality data acquisition, cloud computing and machine learning algorithms that facilitate rapid searching in library databases for spectral and structural similarities (i.e., GNPS, and MotifDB derived from MS2LDA), prediction of molecular formula and structure (SIRIUS + CSI:FingerID), and chemical class annotations (ClassyFire). MolNetEnhancer incorporates GNPS molecular networking, MS2LDA and the in silico annotation tools (ClassyFire, SIRIUS + CSI:FingerID, etc.) outputs, to provide detailed structural and chemical molecular information for large-scale MS data exploration and annotation.

The developments in metabolomics platforms enable large-scale MS/MS experiments containing thousands of distinct spectra from a single sample. These detected features and their MS/MS spectra can be used for matching to spectral libraries and can include indicative fragmentation patterns that provide information on the often yet unknown chemical structures that were fragmented [221,222]. However, this process is still cumbersome and laborious for large-scale data, particularly for plant metabolomics data which is vastly diverse in physiochemical properties [222,223]. Hence, we are currently witnessing the development of tools that can analyze such large-scale, complex metabolomic data using computational networking approaches such as molecular networking (MN), which provides visualization of all the detected features and their chemical relationships by grouping structurally similar features into a network to enable mining of the metabolome (Figure 4) [224,225,226].

#### 2.4.1. Spectral Similarity and Substructure Based Annotation

GNPS, a web-based ecosystem for the sharing, storage and analysis of raw, processed or annotated MS spectral data, enables the generation and analyses of molecular networks (Figure 4) [6,227]. MN through GNPS also enables rapid dereplication, the process of identifying known metabolites, as well as detection of analogs and the discovery of novel metabolites from the acquired MS spectral data [224,228]. Dereplication is achieved by automatically matching the thousands of MS/MS spectra acquired to public MS/MS spectral libraries within GNPS [224,229]. The large-scale spectral matching of MS spectral data is one approach that GNPS uses to facilitate the analysis of vast number of samples, as exemplified by the analysis of 15 batches of Microctis Folium (*Microcos paniculata* L.) leaf extracts that resulted in the identification of 168 metabolites, of which 165 metabolites were identified for the first time in Microctis Folium, and approximately 500 analogues could also be annotated [227]. In addition to GNPS, MS2LDA is another metabolome mining tool that uses an unsupervised Latent Dirichlet Allocation (LDA) algorithm to group molecules based on shared molecular substructures (i.e., as conserved fragments and neutral loss features), referred to as Mass2Motifs, regardless of their overall spectral similarity (Figure 4) [8,230].

The ms2lda.org web application allows us to inspect the extracted Mass2Motifs and annotate them by expert knowledge, comparison of mass fragments and neutral losses to those in MS/MS libraries, or by either comparison to Mass2Motifs extracted from earlier experiments [8,230]. This process is, however, laborious, particularly for large-scale data, and thus MS2LDA has been improved for automating the matching process to include the analysis of multiple samples with MotifSets of structurally characterized Mass2motifs from MotifDB together with “free Mass2Motifs” to capture the yet “unknown” chemistry not captured yet in MotifDB [231]. MS2LDA was applied to MS/MS data of 70 *Rhamnaceae* plant species and resulted in the extraction of 200 Mass2Motifs, of which 25 were annotated with substructures. Characterized Mass2Motifs enabled the probing of substructural diversity within the plant family. For instance, only the Rhamnoid clade was found to develop diversity in flavonoid glycoside, whereas the Ziziphoid clade developed more variety in the triterpenoid pathway [9]. Thus, MS2LDA enables the extraction of substructural diversity within each class of metabolites from complex datasets and provides meaningful biochemical interpretation. Furthermore, an increase in structurally characterized Mass2Motifs will accelerate the identification of metabolites within plants dark matter with high confidence and thereby enhance understanding of plant mechanisms [9,231]. Despite these advances, structural elucidation of entire (plant) metabolites remains challenging in untargeted metabolomics workflows [171]. In the following sections, a number of computational metabolomics tools are highlighted that further enhance the annotation of either complete structures, substructures, or chemical compound class information to (plant) metabolomics profiles.

#### 2.4.2. Structure-Based Annotation

Compound Structure Identification (CSI): FingerID is a web-service tool for searching in molecular structure databases using MS/MS data (Figure 4) [171,232]. CSI:FingerID converts a spectrum into a fragmentation tree that is searched against a database of known trees to predict a molecular fingerprint of the unknown compound. Although CSI:FingerID achieves increased identification rates compared to other related methods, it searches fragmentation trees individually [171,233]. Integration of CSI:FingerID with SIRIUS 4, a software tool for the computational annotation of MS/MS data, enables the full LC-MS/MS dataset to be processed instead of an individual compound [171,234].

The SIRIUS 4 tool offers molecular formula annotation and, when integrated with CSI:FingerID, structure database search and ranked annotations (Figure 4). This is achieved by first analyzing isotope patterns and fragmentation trees to determine the molecular formula of the query compound, followed by the use of CSI:FingerID to predict molecular fingerprint of the resultant spectrum and fragmentation tree. The predicted fingerprint is searched against the structural database to identify the most likely candidate [171,235]. SIRIUS 4 is capable of deducing information of unknown compounds, through Zodiac, now including molecules with higher masses (>500 Da) that are particularly of interest to plant metabolomics researchers, that are not structurally annotated and thus facilitate the mining of the metabolome [194]. Similarity score functions are crucial for the accurate matching of experimentally acquired spectra to library spectra for structural elucidation and for the assessment of spectral pair similarities [236].

#### 2.4.3. Spectral Similarity Scoring for Library Matching and Correlation of Spectra

Spec2Vec, inspired by a natural language processing based algorithm, is a novel spectral similarity score that learns the relations between fragments to enable accurate spectral library matches. Spec2Vec computationally calculates similarity scores more efficiently than cosine-based scores, correlates more closely with structural similarity and identifies relationships between different spectra that are chemically related, especially for larger molecules with two or more subtle local modifications. Spec2Vec thus enables the rapid querying spectra of unknown molecules against all spectra in large databases and the selection of potential candidates for further exploration [236]. Additionally, Spec2Vec will enhance interpretation as it facilitates the correct assignment of metabolites, whose chemical class can be annotated and used to supplement metabolite annotation [237]. Despite its remarkable performance, Spec2Vec was not trained for the task of returning a higher similarity score for spectral pairs of structurally more closely related metabolites; therefore, very recently, MS2DeepScore was introduced that uses a Siamese neural network to predict the structural similarity between two chemical structures solely based on their MS/MS fragmentation spectra [238]. MS2DeepScore outperforms classical spectral similarity measures as well as Spec2Vec in retrieving chemically related compound pairs from large mass spectral datasets, thereby illustrating its potential for spectral library matching.

#### 2.4.4. Chemical Compound Class-Based Annotation

ClassyFire, a web-based program, enables systematic chemical classification into a formal chemical ontology (Figure 4). ClassyFire automatically assigns all known chemical compounds to the predefined taxonomy ChemOnt based on their chemical structures and structural features [239,240]. The ClassyFire server and database consists of more than 77 million compounds that vary from drugs, toxins, phytochemicals, natural and synthetic molecules. Access to this database via the web server enables rapid, large-scale and automated chemical classification that can aid in the annotation or enrichment of known and unknown compounds [237,239,241]. MolNetEnhancer is a software package that incorporates the outputs of GNPS MN, MS2LDA, in silico annotation tools such as SIRIUS+CSI:FingerID, and the automated chemical classification through ClassyFire, into a single workflow (Figure 4) [237].

MolNetEnhancer reveals detailed information on molecular families and the subtle structural differences between them, thus resulting in enhanced molecular networks that facilitate rapid metabolite exploration of large complex datasets by providing a global visual of chemical diversity [226,237]. Another chemical classification tool is NPClassifier, a DL tool for automated structural classification of natural products (NP), that uses chemical fingerprints as input encoding and classifies NP at three levels; 7 Pathways, 70 Superclasses and 653 Classes. NPClassifier has the potential to accelerate NP discovery by enabling large-scale metabolome mining [242,243].

Most recently, the chemical class assignment and ontology prediction using mass spectrometry (CANOPUS) DNN tool for systematic compound class annotation was introduced. CANOPUS predicts compound classes from fragmentation spectra, including biologically relevant classes. CANOPUS specifically targets compounds that have no spectral or structural reference data available and predicts classes for which no MS/MS training data available [241,244]. CANOPUS assigns compound classes to all MS/MS features for which fragmentation trees can be computed in a LC-MS/MS run and automatically provides structural insights of novel compounds from crude samples, thus accelerating the structural elucidation process or allowing prioritization of a subset of metabolite features to further examine [244]. CANOPUS and the aforementioned computational tools and technological advancements are increasingly being applied in metabolomics studies. Whilst there is a lot of promise in these kinds of approaches, exactly how they benefit and improve plant metabolomics workflows is yet to be witnessed. We refer the interested reader to a recent review that further explains the workings of substructure-based, chemical compound class-based, and network-based strategies for metabolite annotation [245].

#### 2.4.5. Large-Scale and Repository-Wide Metabolomics Analyses

The 4IR technologies will improve plant metabolomics workflows and facilitate complex design studies by, for instance, investigating plant response mechanisms to multiple stresses simultaneously. Automation in sample preparation and in analytical platforms, equipped with analytical intelligence will enable large metabolite coverage, rapid, automatic and potentially remote data acquisition from complex samples such as plants. Development and implementation of machine and deep learning algorithms will aid in data mining and pathway modelling by ensuring acquisition of reliable data with minimal noise and artifacts, which relevant features associated to the particular stress are extracted from and confidently annotated through spectral library matches, thus achieving rapid large-scale annotations of metabolites that contribute to elucidating the metabolome studied, in this case the plant metabolome in response to abiotic stresses. The expected transition from experiment-based analysis toward repository-wide analysis has recently gained traction by the introduction of the MASST [246] and ReDU [247] frameworks that allow researchers to locate similar MS/MS spectra across all public datasets, i.e., akin to doing a BLAST search with a genetic sequence. An increasing number of these public datasets are annotated with consistent metadata through a controlled vocabulary (i.e., ReDU) or ontologies (i.e., MetaboLights [7]). Thus, if a researcher finds hits across different public datasets, the metadata (sample information) can provide valuable information on the possible source of that fragmented component in their sample. Moreover, plant researchers can easily select subsets of publicly available datasets of the plant-based origins relevant to their experiment to compare their data to and facilitate the annotation of plant metabolites.

## 3. Metabolomics and Plant Responses to Abiotic Stresses—Current Frameworks

Abiotic stresses are changes in the environmental conditions that negatively affect plants’ growth and development, metabolism and physiology. The main abiotic stresses include extreme temperatures, drought, waterlogging, light, radiation, salinity, heavy metals and chilling [14,18]. Abiotic stresses activate complex cellular responses that are increasingly being elucidated by progresses made in exploring and understanding plant responses to abiotic stress factors at the whole-plant, physiological, cellular, biochemical and molecular levels. As briefly mentioned in Section 1.2, plant responses to abiotic stress involve the activation of multi-layered events comprising complex gene interactions and crosstalk with different molecular pathways and networks. Current knowledge (and understanding) of plant responses to abiotic stresses (Section 1.2) is still the tip of an iceberg. Comprehensive and accurate metabolic descriptions (models) are imperatively needed and essential for devising a roadmap for the next generation of crops resilient to environmental deterioration.

As mentioned in Section 1.1, metabolomics is increasingly enabling the decoding of the language of cells at molecular level, advancing the understanding of regulatory network rules and mechanistic events at cellular and chemical space of the plant under consideration. The application of this multidisciplinary *omics* science in studying plant responses to abiotic stresses has gained a momentum, generating insights into molecular events that govern plant ‘defensome’. To illustrate such applications, in this review, we focus on drought and salinity stress conditions. Drought and salinity are the two most impactful abiotic stresses, affecting 45% and 19.5% of the world’s agricultural lands, respectively. These two (globally) major abiotic stresses (drought and salinity) lead to an annual economic loss above USD 20 billion in developing countries [248]. While drought stress is caused by water deficit, salinity is the result of accumulation of ions (sodium and chloride) in the rhizosphere [248,249,250]. Both stresses affect morphological, physiological and biochemical processes in plants, resulting in growth suppression and, failing stress alleviation, eventually in cell death. The onset of drought and salt stresses in plants prompts stress-responsive mechanisms including the signal perception and transduction via several signalling pathways including second messenger signalling (ROS, Ca^2+^), the kinase-signalling cascades (MAPK) and hormone signalling [251,252,253]. Stress signal perception triggers several stress-responsive processes encompassing genetic reprogramming, activation of ion channels and synthesis of osmolytes.

Several metabolic studies on different plants have indicated altered primary and secondary metabolite profiles in response to salinity and drought stresses (Table 3). Sugars, amino acids, and organic acids and their derivatives are the most altered primary metabolites in osmotic stressed plants [254,255,256]. Additionally, the perturbed metabolism of secondary (specialized) metabolites (phenolics acids, flavonoids, phytohormones) has also been detected in several metabolic studies in response to salinity and drought stresses [257,258,259]. Comprehensive characterisation of stress-responsive metabolic profiles and fluxes is essential for the elucidation of the molecular mechanisms used by plants to combat abiotic stress conditions. Such knowledge can be translated into suitable biotechnological tools for the development of stress-tolerant crop plants for sustainable food security.

Metabolomics was applied to elucidate the integrative biochemical networks of two spring-wheat cultivars (*Bahar*—drought-susceptible; *Kavir*—drought-tolerant) to drought stress. Metabolome changes of wheat leaves exposed to 7 days of drought stress was investigated with LC-MS. Three hundred peaks were detected per sample, with 165 and 146 identified metabolites for *Bahar* and *Kavir*, respectively. The main metabolites changed due to drought stress were amino acids, organic acids and sugars. In *Bahar*, proline, methionine, arginine, lysine, aromatic and branched chain amino acids were increased. In contrast, only the purine metabolic pathway was significantly affected by the drought stress in *Kavir*. Metabolomics in this study thus provided better understanding in the wheat plant response mechanisms to drought stress [256].

Additionally, metabolomics has also been used to investigate two foxtail millet (*Setaria italica*) cultivars of Yugu2 and An04 subjected to salinity stress. A total of 720 metabolites were identified from the LC-MS data, with 150 metabolites involved in the response to salt stress, ranging from organic acids, sugars, phenolics, amino acids and others. The flavonoids were significantly up-regulated in the roots, with the majority of them and other secondary metabolites being observed in Yungu2. This metabolomics analysis revealed the flavonoid pathway, starch and sucrose metabolism, glutathione metabolism, glycophospolipid metabolism, ascorbate and aldarate metabolism, phenylpropanoid and shikimate pathways to be involved in the responses to salinity stress. The authors also noted metabolites such as lysophospholipids, which play a role in response to salinity stress. Therefore, they suggest that there are diverse existing or new response mechanisms in foxtail millet to cope with salinity stress [260].

## 4. Conclusions and Perspectives

Metabolomics has been crucial in elucidating cellular and molecular mechanisms of plant-environmental interactions. There are still some bottlenecks that limit or hamper the comprehensiveness of biological insights derived from this multidisciplinary omics science, metabolomics. Some of these challenges include the complexity, non-linearity and volume of the generated (metabolomics) datasets, the lack of accurate metabolite annotation, the difficulty in assigning precursor ions to product ions for overlapping chromatographic peaks, and the lack of large-scale analysis tools and reconstruction of metabolic pathways. These challenges can be addressed by the implementation or a revival of 4IR technologies in plant metabolomics workflows. For example, the automation of sample preparation methods has reduced the sample preparation time with minimum variation and human error, thereby resulting in large-scale metabolite extraction, increased metabolome coverage and the potential to discover novel metabolites that will illuminate pathways involved in plant response molecular and cellular events, thereby elucidating plant response mechanisms. Furthermore, the advancement and incorporation of analytical intelligence in instruments facilitates accurate quantification of metabolites through automatic optimization of chromatographic conditions to enhance peak picking and resolution, and reproducibility of the data for accurate and confident metabolite annotation. Machine and deep learning algorithms can handle non-linear data, thus accurately identifying complex relationships within plant data, and extracting relevant metabolic features that correlate to biological origin (i.e., abiotic stress) without bias or approximation. Machine learning and computational tools with predictive capabilities and rapid search of unknown metabolites in databases aid in metabolic pathway and molecular formula predictions, and rapid metabolite annotation and/or structural elucidation at various levels from chemical compound class, via substructures, to complete metabolite structures, thus facilitating comprehensive large-scale mining of (plant) metabolomes.

We expect that future developments of 4IR technologies in plant metabolomics will further increase automation and connectivity within workflows. For instance, robotic systems may link prepared samples from the automated sample preparation method instruments to the analytically intelligent instruments with a custom software that utilizes the internet of things and wireless telecommunication technologies to automatically upload the resultant data onto a cloud server for download or sharing by the user, thus enabling remote data acquisition and analysis. Additionally, portable analytical devices that facilitate metabolite data acquisition directly from the plant tissue without extraction, will be developed and may be mounted on to drones for field purposes. An increase in novel computational tools for processing and analysis of spectral imaging data is anticipated. Development of computational tools based on machine and deep learning algorithms for predicting and grouping metabolite functional activity will enable pathway constructions and elucidate/predict the synergistic behavior of metabolites/pathways in response to the changing environment, thus further elucidating plant response mechanisms. The integration of 4IR technologies will also facilitate rapid data analysis. For instance, the integration of analytical intelligent instruments with cloud servers, computational tools and algorithms, will enable simultaneous (i.e., real time) metabolite acquisition and metabolite annotation, thus enhancing rapidity of plant metabolomic studies and rapid elucidation of plant response mechanisms to stress. The first examples of tools that are the building blocks of such large-scale analyses were highlighted in this review. From our review, it becomes clear that in the last two decades, metabolomics workflows have matured and that we are at the dawn of a new era where computational metabolomics analysis workflows in combination with more automated data sampling and acquisition will decrease the barrier to implement metabolomics studies in research programs in academia and industry. We believe that this will increase the rate of development for next generation of crops that are highly productive and resilient to climate change.

## Figures and Tables

**Table 1:** Automated sample preparation methods with their advantages and disadvantages over traditional sample preparation methods.

Method	Description	Advantages	Disadvantages	Originally Automated? (Yes/No)	Reference(s)
Solid-phase extraction (SPE)	Extracts metabolites based on their chemical and physical properties that determine their distribution between the mobile liquid phase and a solid stationary phase. Targeted metabolites are released from stationary phase by changing the mobile phase into the elution solvent.	Enhanced selectivity, rapid, reproducible and economical.	Poor metabolite coverage	No	[43,44,45,46,47]
Solid-phase microextraction (SPME)	Extracts a range of metabolites from a variety of matrices by the insertion of a polymer-coated fiber into either the vial headspace, liquid sample or exposed in vivo. The metabolites diffuse from the sample onto the fiber.	Enhanced sensitivity, minimum invasiveness, enhanced analysis throughput and compatibility with in vivo sampling and extraction.	Time-consuming steps in equilibration of the fibre (30 min) and sample extraction (up to 5 min), low metabolite coverage, and its expensive.	Yes	[40,48,49,50]
Dispersive liquid–liquid microextraction (DLLME)	Extraction solvent (i.e., water-immiscible organic solvent) is added to a dispersive solvent (i.e., water-miscible solvent), the mixture is then injected into the sample to form a homogenous solution. Induced dispersion increases surface contact between extract and the sample, thus resulting in instantaneous extraction.	Simple, cost-effective, rapid, has high extraction recovery, reduced solvent consumption and has high reproducibility.	Uses halogenated and organic solvents, requires manual/mechanical agitation of the sample for dispersion of the organic solvents in the sample solution, and time-consuming phase separation step.	No	[48,51,52]
Electromembrane extraction (EME)	An electrical field is applied between the sample and the acceptor compartments, separated by a membrane of organic solvent (i.e., the support liquid membrane (SLM)). Charged ionic metabolites are extracted from the sample solution, through the SLM, and into the acceptor compartment. Proteins, salts, etc. are incapable of passing the SLM, thus metabolites are recovered in the aqueous phase.	Enables large-scale automation.	Difficulty of extracting hydrophillic metabolites.	No	[48,52,53]
Hollow fiber liquid–liquid microextraction (HF-LLME)	Two-phase mode: The hollow fiber (HF) is soaked with the extraction solvent and exposed to the sample’s solution or headspace, an equilibrium between solvent and sample is establishes, thus resulting in the extraction of metabolites from the sample into the solvent. Three-phase mode: The center of the HF contains an aqueous phase (i.e., acceptor phase), in addition to the soaked HF pores with organic solvent. The HF is exposed to the sample where two equilibriums are established. The first is between the sample and the solvent, followed by the second between the solvent and the acceptor phase, thus metabolites are extracted from the sample into the acceptor phase of the HF through the solvent.	Highly selective and concentrate metabolites.		No	[51,52]
Single drop microextraction (SDME)	Similar to HF-LLME. A syringe is used instead of a HF and only a drop of the extractant solvent is required.	Simple, cost-effective and time-saving.	Limited by partial solubility of organic solvents in water, limited extraction volume, metabolite losses due to volatility and dislodgement of the extractant solvent.	No	[51,52,54]
Accelerated solvent extraction (ASE)/Pressurized liquid extraction (PLE)	The solvent’s temperature is elevated beyond its boiling point to increase its solubilizing capacity and reduce its viscosity to penetrate into the sample matrix and increase the metabolites’ diffusion rate. Additionally, the elevated pressure ensures the solvent remains in the liquid phase and aids it in penetrating through the sample matrix, which maximizes solvent and metabolite contact, and result in effective extraction.	Reduced solvent usage and rapid.		No	[42,55,56]
Supercritical fluid extraction (SFE)	Utilizes gas properties above their critical points as solvents to facilitate the extraction of non-polar to semi-polar metabolites from plant materials.	Enhanced sensitivity and accuracy, reduced extraction time, ideal for thermo-labile metabolites and reduced use of organic solvents.	Very expensive.	Yes	[42,56,57,58]
Microwave-assisted extraction (MAE)	Microwave, electromagnetic radiation with a frequency in the 0.3–300 GHz range, energy is used to extract polar metabolites from plant materials by heating the solvent.	Reduced extraction time (15-20 min), reduced solvent consumption, improved extraction yield and precision.	Operates at relatively high temperature which is problematic for thermally liable metabolites, low extraction yield for non-polar solvents and requires a centrifugation step to remove solid materials from extractant.	No	[57,59,60]
Ultrasound-assisted extraction (UAE)	Utilizes ultrasonic energy and solvents to extract secondary metabolites from various plant materials.	Reduced extraction time, solvent consumption, energy, thermal degradation, extraction temperature and equipment size, enhanced mass transfer, extraction yield and high extract recovery.	Low extraction efficiency.	No	[57,61,62,63]

**Table 2:** Selected recent metabolomics studies showcasing 4IR technologies, particularly the use of ML methods in plant research.

Metabolomics Study	ML Method ^1^	Reference
ML-modelling for prediction of metabolic pathways of plant enzymes.	SVM, ANN, NB, DTC	[160]
Identification of central and predictive molecular components of plant metabolic stress response.	SVMs, NNC, DTC	[157]
Untargeted metabolomics to reveal diversity of the metabolome in seeds of *Camelina* species.	DL, ANN	[155]
Characterisation of adaptive and signalling responses based on metabolite content under abiotic stresses.	SVM, DPClus	[148]
Detection of aflatoxin metabolite in chilli pepper using machine vision.	SVM, RF	[158]
Detection and resolution on plant metabolites (*S.lycopersium*) using mass spectrometry imaging.	DCNN	[156]
Discovery of Q-markers from Jinqi Jiangtang for medicinal purposes.	ANN	[154]
Prediction of metabolic pathways in correlation networks in the pericarp of a tomato.	ML algorithms	[161]
Discovery and identification of biomarkers using ML algorithms in metabolomic studies.	ANN, DL	[153]
Enhancement of plant metabolite fingerprinting using ML methods.	SVM, RF	[164]

^1^ SVM, Support Vector Machines; ANN, Artificial Neural Networks; NB, Naïve Bayes; NNC, Nearest Neighbour Classifiers; DTC, Decision Tree Classifiers; DL, Deep Learning; RF, Random Forest; DCNN, Deep Convolution Neural Networks.

**Table 3:** An overview of metabolism alterations in response to drought and salinity stresses, and their corresponding roles in plant stress responses.

Metabolite Group	Stress-Responsive Roles	Plant Species	References
Amino acids	ROS scavenging (proline), protein stabilisation and synthesis, redox control	*Dianthus superbus, Lens esculenta*	[261,262]
Polyols	Protection of photosynthesis systems, ROS scavenging, protein stabilisation	Rice, apple leaves, *Fraxinus excelsior,* *Zea mays*	[263,264,265,266]
Organic acids	Energy production, signalling molecules, antioxidant activities	*Oryza sativa*, Wheat	[249,267]
Sugars	Signalling molecules, carbon energy reserve, maintenance of redox homeostasis, osmoprotectants	*Solanum* *lycopersicum, Triticum aestivum*	[268,269]
Polyamines	Activation of antioxidant enzymes, regulation of ion channels activity, protein and membrane stabilisation	Tobacco, *Triticum aestivum*	[270,271]
Phenolics	Hormonal regulation, antioxidant activity, photosynthetic activity	*Patagonian shrublands, Amaranthus tricolor*	[272,273]
